# Iodine status of breastfed infants and their mothers' breast milk iodine concentration

**DOI:** 10.1111/mcn.12993

**Published:** 2020-03-11

**Authors:** Erna Petersen, Birna Thorisdottir, Inga Thorsdottir, Geir Gunnlaugsson, Petra Arohonka, Iris Erlund, Ingibjörg Gunnarsdottir

**Affiliations:** ^1^ Department of Clinical Nutrition Landspitali University Hospital Reykjavik Iceland; ^2^ Unit for Nutrition Research, Faculty of Food Science and Nutrition, Landspitali University Hospital University of Iceland Reykjavik Iceland; ^3^ School of Health Sciences University of Iceland Reykjavik Iceland; ^4^ Faculty of Sociology, Anthropology and Folkloristics, School of Social Sciences University of Iceland Reykjavik Iceland; ^5^ Forensic Toxicology Unit, Biochemistry Laboratory Finnish Institute for Health and Welfare (THL) Helsinki Finland

**Keywords:** diet, human, infant, iodine, lactation, milk

## Abstract

Iodine is an essential nutrient for growth and development during infancy. Data on iodine status of exclusively (EBF) and partially breastfed (PBF) infants as well as breast milk iodine concentration (BMIC) are scarce. We aimed to assess (a) infant iodine nutrition at the age of 5.5 months by measuring urinary iodine concentration (UIC) in EBF (*n* = 32) and PBF (*n* = 28) infants and (b) mothers' breast milk iodine concentration (*n* = 57). Sixty mother–infant pairs from three primary health care centres in Reykjavik and vicinities provided urine and breast milk samples for iodine analysis and information on mothers' habitual diet. The mother–infant pairs were participants of the IceAge2 study, which focuses on factors contributing to infant growth and development, including body composition and breast‐milk energy content. The median (25th–75th percentiles) UIC was 152 (79–239) μg/L, with no significant difference between EBF and PBF infants. The estimated median iodine intake ranged from 52 to 86 μg/day, based on urinary data (assuming an average urine volume of 300–500 ml/day and UIC from the present study). The median (25th–75th percentiles) BMIC was 84 (48–114) μg/L. It is difficult to conclude whether iodine status is adequate in the present study, as no ranges for median UIC reflecting optimal iodine nutrition exist for infants. However, the results add important information to the relatively sparse literature on UIC, BMIC, and iodine intake of breastfed infants.

**Key messages**

- Iodine status of breastfed infants in Iceland was unknown prior to this study.
- The median (25th–75th percentiles) UIC and BMIC was 152 (79–239) and 84 (48–114) μg/L, respectively, with no significant difference between EBF and PBF infants.
- The study adds important information to the relatively sparse literature on infant UIC and BMIC.

## 1 INTRODUCTION

Iodine is essential for production of thyroid hormones that are crucial for growth and numerous processes of neural and cognitive development (Bougma, Aboud, Harding, & Marquis, 2013; Leung, Pearce, & Braverman, 2011; Zimmermann, Jooste, & Pandav, 2008). Structural brain changes start in foetal life, involving neuronal proliferation and development of axons, synapses, and myelination, and continue well after birth. The plasticity of the central nervous system in the first years of life makes it especially vulnerable to external influences such as nutritional deficiencies and excesses. Thyroid hormone production rates are higher in infancy than any other life stage. Consequently, iodine deficiency can have detrimental effects in the prenatal stage, when cortical formation is at its peak, and postnatally due to neuronal plasticity (Berbel et al., 2009; Delange, 2007; Gothié, Demeneix, & Remaud, 2017; Horn & Heuer, 2010; Leung et al., 2011; Rovet, 2014; Trumpff et al., 2013; Zimmermann, 2007, 2011, 2012). The mother is the foetus' only source of thyroid hormones during the first half of pregnancy until its own thyroid starts functioning in the second half. From then on and during exclusive breastfeeding (EBF), the infant relies solely on the mother for iodine to produce its own thyroid hormones.

In the past, iodine status in Iceland has been found to be optimal among adults, adolescents, and pregnant women (Gunnarsdottir et al., 2010; Gunnarsdottir et al., 2013; Gunnarsdottir & Dahl, 2012; Gunnarsdottir, Gustavsdottir, & Thorsdottir, 2009; Nyström et al., 2016). The main reason was the relatively high intake of fish and dairy, together providing 76% of the total iodine intake in the adult Icelandic diet (Þorgeirsdóttir et al., 2011). The dietary habits of the Icelandic population are, however, changing. As a consequence of decrease in fish and dairy intake, insufficient iodine status was, in fact, recently observed in a cohort of pregnant women in Iceland (Adalsteinsdottir et al., 2020). Breastfeeding is highly prevalent in Iceland compared with other high‐income countries, with 85% of infants EBF in their first week of life and 35% still EBF at 5 months of age (Hörnell, Lagström, Lande, & Thorsdottir, 2013; Sigbjörnsdóttir & Gunnarsdóttir, 2012; Thorisdottir, Thorsdottir, & Palsson, 2011). No recommendations exist for optimal iodine status of infants, but a cut‐off of UIC >100 μg/L for sufficiency for children <2 years has been proposed, based on the criteria used for older children (Andersson, de Benoist, Delange, & Zupan, 2007; WHO/UNICEF/ICCIDD, 2007). Furthermore, no official guidelines for the cut‐off for median breast milk iodine concentration (BMIC) exist, but a concentration of >75 μg/L has been suggested to be adequate (Azizi & Smyth, 2009). It should be noted that studies from iodine sufficient areas (indicated by median UIC > 100 μg/L in school‐aged children and adults) have reported median values that are clearly higher, that is, BMIC 150 to 180 μg/L (Delange, 2007; Dorea, 2002; Semba & Delange, 2001). The recommended iodine intake of children <2 years of age is 90 μg/day (WHO/UNICEF/ICCIDD, 2007).

Most of the previous studies on UIC in infancy and BMIC have been conducted in areas of deficiency (Andersen, Moller, & Laurberg, 2014; Dold et al., 2016; Henjum et al., 2017; Jorgensen, O'Leary, James, Skeaff, & Sherriff, 2016; Mulrine, Skeaff, Ferguson, Gray, & Valeix, 2010; Skeaff et al., 2005; Stinca et al., 2017) and in areas where intakes are excessive or borderline excessive (Aakre et al., 2016; Nepal et al., 2015; Osei et al., 2016). Only few published studies report UIC from areas of sufficiency (Gordon et al., 2014; Leung et al., 2012; Yang et al., 2014). Data on iodine status of EBF and PBF infants between 5 and 6 months of age are generally lacking as is data on iodine concentration in breast milk from mothers of infants of that age.

The aim of this study was to assess infant iodine nutrition at the age of 5.5 months, by measuring infant UIC in EBF and partially breastfed (PBF) infants as well as BMIC.

## 2 METHODS

Sampling of biomarkers and collection of other relevant data for the present study were part of the IceAge2 study (Growth and Body Composition in Breastfed Infants: Study on Age of Introduction of Complementary Foods in Iceland). IceAge2 is a prospective cohort study with the aim of investigating breast milk and breastfeeding among infants who are exclusively and partially breastfed at nearly 6 months in terms of characteristics that are hypothesized to contribute to growth and development of body composition in infancy. The focus is on specific novel outcomes and factors including body composition development, breast‐milk metabolizable energy and hormone content, and the role of infant temperament and appetite.

### 2.1 Subjects

Mother–infant pairs were recruited during routine postnatal visits at infant age 5 months in two primary health care centres in Reykjavik and one in the capital vicinity. The eligibility criteria were Icelandic mother, singleton birth, gestational age 37–42 weeks, birth weight > 2,500 g, EBF >2 months, no diseases or defects likely to affect growth or body composition, and either EBF from birth until at least 5.5 months (EBF group) or PBF from 3 to 5 months and receiving at least 100 g or ml of complementary foods in addition to breast milk at 5.5 months (PBF group). All mothers provided written informed consent.

### 2.2 Study design and sampling methods

Urine and breast milk samples were collected between December 2014 and June 2017. Mothers collected a single 2‐ml spot urine sample from their infants at 5.5 months (25 weeks) by placing four to five absorbent cotton pads in infant's diaper. When the infant had wet the diaper, urine was pressed out of the cotton pads into 2‐ml vials using 20‐ml disposable syringes. Dorey and Zimmermann (2008) have previously described a similar non‐invasive method for collecting infant urine samples for analysis of iodine content by using sterile pad inserts for diapers and the validation of the method. We measured possible iodine content of the cotton pads and possible entrapment of iodine in cotton fibres, as described below. Mothers then collected a 10‐ to 15‐ml sample of breast milk at the start of a single feed (fore milk) into a 100‐ml container. Breast milk was stored together with the urine sample in household fridges until collected by research staff. The research staff transferred the collected breast milk into 2 mL vials. The samples were then frozen at −80°C at Landspitali University Hospital in Reykjavík, Iceland. Information on sociodemographic characteristics was collected, including marital status, maternal and paternal education, and parity and information on supplement and medication use and whether or not mothers ate from all food groups. Mothers also answered a food frequency questionnaire, providing information on their habitual consumption of key iodine containing foods.

### 2.3 Iodine concentration and estimated intake

When all urine and breast milk samples had been collected, they were transported on dry ice to Helsinki, Finland. Samples were assayed at the National Institute for Health and Welfare. Iodine concentration of the samples was measured by inductively coupled plasma mass spectrometry (ICP‐MS), using an Agilent 7800 ICP‐MS system (Agilent Technologies Inc., Santa Clara, CA, USA). This is the recommended method for the determination of trace elements in urine and more complex matrices like breast milk because of its specificity, sensitivity, and low detection limits (Dold et al., 2016). For iodine measurements, 100 μl of urine/breast milk sample was extracted by using 2% (*w*/*v*) ammonium hydroxide. Iodine was scanned on *m/z* = 127 and Tellurium was used as an internal standard. All samples were analysed in duplicate.

Iodine contamination tests on the cotton pads used to collect urine during the study period were performed as well as recovery tests (all three batches). Ultrapure water and control urine (with known iodine concentration) were added to the cotton samples. For the purpose of accuracy, the sampling protocol in IceAge2 study required that parents check their child's diaper frequently every 30 min. This means that urine was aimed to be in the cotton pads for a maximum of 30 min. Therefore one half of the control urine samples waited for 0–3 min in the cotton pads and the other for 30–33 min. The test using ultrapure water revealed iodine concentrations below the detection limit of the ICP‐MS method, which is 2 μg/L. The test using control urine showed normal and accurate iodine concentrations of urine samples. Therefore, the cotton pads were considered a reliable method to collect urine in our study. Infant iodine intake was estimated based on UIC and assuming an average urine volume of 300–500 ml/day (Andersson et al., 2007) and compared with recommended iodine intake of 90 μg/day for children <2 years (WHO/UNICEF/ICCIDD, 2007). Infant iodine intake was also estimated using estimated breast milk consumption of 5‐ and 6‐month‐old infants (796 and 854 ml/day, respectively; World Health Organization [WHO], 2002).

### 2.4 Statistical analysis

Data were analysed using IBM SPSS for Windows, version 24 (IBM Corp., Armonk, N.Y., USA). Normally distributed data were presented as means (standard deviation), but other data were reported as medians (25th–75th percentile) or numbers (%). UIC and BMIC are presented as medians (25th–75th percentile). Spearman's rho was used to assess possible correlation between UIC, BMIC, and maternal intake of dietary sources of iodine (dairy and fish). Student's *t* test was used to compare means and Mann–Whitney U test was used to test for differences between groups when data was non‐parametric. Pearson Chi‐square test and Fisher's exact test were used on categorical data.

### 2.5 Ethical considerations

The study was approved by the National Bioethical Committee (VSN 13‐146) in Iceland. Additional approval was granted for the analysis of UIC and BMIC (VSN 13‐146‐V3 and V6).

## 3 RESULTS

Sixty mother–infant pairs participated in the study, thereof 32 in the EBF group and 28 in the PBF group. Characteristics of mothers and infants are shown in Table 1. Majority of the mothers had older children (60%) and were married or cohabiting (97%). No significant difference was found between characteristics of infant–mother pairs based on infant feeding methods (EBF vs. PBF).

**Table 1:** Characteristics of mothers and their breastfed infants

Characteristic	EBF infants (*n* = 32)	PBF infants (*n* = 28)	*p* value
Infants			
Age at sampling (weeks)^a^	25.0 (0.6)	25.1 (0.7)	.4^c^
Birth weight (kg)^a^	3.9 (0.5)	3.6 (0.2)	.3^c^
Gestational age (completed weeks)^a^	40 (1.4)	40 (0.9)	1.0^c^
Boys^b^	14 (43.8)	17 (60.7)	.2^d^
Weight at sampling (kg)^a^	7.9 (0.9)	7.7 (0.9)	.8^c^
Mothers			
Age (years)^a^	30.2 (4.4)	30.6 (5.3)	.8^c^
Parity^a^			.1^d^
Primiparous^b^	10 (31.3)	14 (50.0)	
Multiparous^b^	22 (68.8)	14 (50.0)	
Delivery by caesarean section^b^	6 (20.0)	6 (21.4)	1.0^e^
Body mass index^a^	25.4 (5.0)	27.3 (5.6)	.2^c^
Use of supplements	23 (72)	24 (86)	1.0^e^
Use of iodine containing supplements^b^	6 (19.4)	4 (14.3)	.7^e^
Mothers who exclude fish and dairy^b^	1 (3.6)	2 (7.1)	1.0^e^
Married or cohabiting^b^	31 (96.9)	27 (96.4)	1.0^e^
Highest completed education^b^			.1^d^
Elementary education or technical/high school	6 (20.0)	10 (35.7)	
University BSc/BA/BEd	10 (33.3)	12 (42.9)	
University MSc/MA/Med or doctorate	14 (46.7)	6 (21.4)	

Abbreviations: EBF, exclusively breastfed; PBF, partially breastfed.

a Data presented as means (*SD*).

b Data presented as *n* (%).

c Statistical difference tested with *t* test.

d Statistical difference tested with Pearson Chi‐square test.

e Statistical difference tested with Fisher's exact test.

All 60 mothers provided spot urine samples from their infants. The median (25th–75th percentiles) UIC was 152 (79–239) μg/L. No difference was seen between feeding groups (158 (97–278) μg/L in the EBF group versus 146 (75–239) μg/L in the PBF group, *p* = .5). Based on the UIC, estimated median iodine intake of the infants in the present study was found to be 52–86 μg/day (Andersson et al., 2007).

Fifty‐seven mothers provided breast milk samples. The median (25th–75th percentiles) BMIC was 84 (48–114) μg/L. No difference was seen between feeding groups (84 [48–120] μg/L in the EBF group vs. 89 [47–111] μg/L in the PBF group, *p* = .9). Using average breast milk consumption of 5‐ to 6‐month‐old infants (WHO, 2002), the estimated iodine intake was found to be 67–71 μg/day. A significant correlation was seen between BMIC and UIC (*r* = .45, *p* < .001).

Median maternal intake of fish and dairy, the two main dietary sources of iodine in the Icelandic diet, was 1.2 times per week and 1.2 times per day, respectively. Table 2 shows median UIC, median BMIC, and infant iodine intake estimated by using UIC and BMIC, in categories of maternal adherence to Icelandic food based dietary guideline on fish (≥two portions per week) and diary (≥two portions per day) intake. More frequent intake of diary, products was associated with higher BMIC (*r* = .36, *p* < .008). Other associations between maternal intake and UIC or BMIC were not found.

**Table 2:** Median UIC, median BMIC, and estimated median infant iodine intake according to maternal adherence to Icelandic food based dietary guideline on fish (≥two portions per week) and diary (≥two portions per day) intake

Iodine source	Maternal intake	*n*	UIC μg/L	BMIC μg/L	Iodine intake based on UIC μg/day^a^	Iodine intake based on BMIC μg/day^b^
Dairy	<two portions per day	38	146	78	49–81	62–67
	≥two portions per day	18	177	99	59–98	79–85
Fish	<two portions per week	44	147	83	49–81	66–71
	≥two portions per week	12	175	96	58–97	77–82

Abbreviations: BMIC, breast milk iodine concentration; UIC, urinary iodine concentration.

a Assuming urine volume of 300–500 ml/day and the UIC analysed in the present study (WHO/UNICEF/ICCIDD, 2007).

b Assuming average breastmilk consumption of 796 and 854 ml/day by 5‐ and 6‐month‐old infants, respectively (WHO, 2002).

## 4 DISCUSSION

The median UIC of infant in the present study of 152 μg/L seems to indicate sufficiency in the population studied when compared with proposed cut‐off for median UIC >100 μg/L for children <2 years (WHO/UNICEF/ICCIDD, 2007). However, this cut‐off is based on criteria for assessing iodine nutrition of older children (WHO/UNICEF/ICCIDD, 2007), and currently, no ranges for median UIC reflecting optimal iodine nutrition exist for infants. Estimated median iodine intake of the infants in the present study was 52–86 μg/day based on UIC and 67–71 μg/day based on BMIC. When compared with the recommended iodine intake of children <2 years of 90 μg/day (WHO, 2007), insufficient iodine intake cannot be excluded.

While most previous studies on infant UIC have either been done in areas of deficiency, reporting considerably lower UIC than in the present study (Mulrine et al., 2010; Stinca et al., 2017) or from areas of excess, reporting higher UIC than in the present study (Aakre et al., 2016; Nepal et al., 2015; Osei et al., 2016), there are at least two studies that can be found in the literature from sufficient areas (Gordon et al., 2014; Leung et al., 2012). In both studies, the UIC was found to be higher than in the present study; the 39 EBF infants (mean age 2.1 ± 0.2 months) in the study of Gordon et al. (2014) had a median UIC 204 μg/L (62–396), and the 64 EBF or PBF infants (mean age 1.6 ± 0.5 months) in the study of Leung et al. (2012) had a median UIC 197 (40–785) μg/L. Leung et al. (2012) reported no information on maternal dietary sources of iodine, but Gordon et al. (2014) reported maternal use of iodized table salts, kelp, iodine containing supplements, and recent consumption of common iodine containing foods. Although historically, Iceland has been known to be an iodine‐sufficient population, recent data suggest insufficient iodine status in pregnant women in Iceland where median UIC was found to be 89 μg/L (Adalsteinsdottir et al., 2020). Median maternal intake of fish (1.2 times per week) and dairy products (1.2 portions per day) in the present study was similar to intake reported by pregnant women in the study by Adalsteinsdottir et al., (2020) Therefore, it is possible that maternal iodine status in the present study might have been insufficient.

Studies have shown considerable variation in BMIC within and between individuals, and although no official guidelines exist for the cut‐off for median BMIC, >75 μg/L has been suggested as adequate (Azizi & Smyth, 2009). However, higher cutoffs have been proposed (Fisher, Wang, George, Gearhart, & McLanahan, 2016), and studies from areas of sufficiency have reported median BMIC 150 to 180 μg/L (Delange, 2007; Dorea, 2002; Semba & Delange, 2001). The results of the present study suggest that a median BMIC of 84 μg/L might not be sufficient for 5–6 months EBF and PBF infants.

Fifty‐three mothers in the present study answered a food frequency questionnaire with semiquantitative response options for dairy and frequency options for fish as a main meal. The median consumption frequency was 1.2 meals a week of fish and 1.2 portions a day of dairy. Although food based dietary guidelines for fish (two times per week) and dairy intake (two portions per day) are not in general based on estimated iodine requirements (Nordic Council of Ministers, 2014; Ólafsdóttir et al., 2014), the results of the present study suggest that adherence to the food‐based dietary guidelines during lactation is likely to result in higher BMIC and UIC of the infants. Dietary intake of lactating women is seldom reported in studies reporting BMIC or UIC of breastfed infants.

The study has several strengths. First, it is the first one to assess infant iodine nutrition in Iceland, breastfed or otherwise. Internationally, most previous studies have assessed iodine nutrition of infants at a younger age or not reported results based on infant feeding methods. Second, the participants were a well‐defined sample of infants of the same age (25 weeks). Third, the sample also had an almost equal number of EBF and PBF infants making comparison between the groups easy. Finally, the analysis of UIC and BMIC were done using the ICP‐MS method, which is considered the best available method for analysing iodine in urine and breast milk. Despite its considerable strengths, there are some limitations. First, the study is limited by its small sample size regarding the questions on iodine status. A larger sample size would have made comparison between groups possible, based on the consumption of dietary iodine sources and use of iodine containing supplements. Second, recruiting of participants was dependent on recruitment for IceAge2, in which evaluation of the iodine status was not one of initial objectives. Therefore, the results may not be extrapolated as being valid for the general public.

This is the first study assessing iodine status of breastfed infants in Iceland. It provides new insights into iodine nutrition of infants that have been exclusively or PBF since birth until they are 25 weeks of age or 6 months old. It is difficult to conclude whether iodine status is adequate in the present study, as no ranges for median UIC reflecting optimal iodine nutrition exist for infants. However, the results add important information to the relatively sparse literature on UIC, BMIC, and iodine intake of breastfed infants.

## CONFLICTS OF INTEREST

The authors declare that they have no conflicts of interest.

## CONTRIBUTIONS

IT, GG, IG, and BT contributed to the design of the study, and BT collected the data together with EP and PA, and IE provided the facilities, reagents, and tools for analysing the collected samples and supervised sample analysis. EP analysed the data and wrote the first draft of the manuscript together with IG. All authors contributed to and critically reviewed the manuscript. They have all approved the final manuscript.
